# Survival of tumor cells after proton irradiation with ultra-high dose rates

**DOI:** 10.1186/1748-717X-6-139

**Published:** 2011-10-18

**Authors:** Susanne Auer, Volker Hable, Christoph Greubel, Guido A Drexler, Thomas E Schmid, Claus Belka, Günther Dollinger, Anna A Friedl

**Affiliations:** 1 Department of Radiation Oncology, Ludwig-Maximilians-Universität München, Germany; 2 Institute for Applied Physics and Metrology, Universität der Bundeswehr München, Neubiberg, Germany; 3 Department of Radiation Oncology, Technische Universität München, Germany

**Keywords:** laser acceleration, proton therapy, dose rate effects

## Abstract

**Background:**

Laser acceleration of protons and heavy ions may in the future be used in radiation therapy. Laser-driven particle beams are pulsed and ultra high dose rates of >10^9 ^Gy s^-1^may be achieved. Here we compare the radiobiological effects of pulsed and continuous proton beams.

**Methods:**

The ion microbeam SNAKE at the Munich tandem accelerator was used to directly compare a pulsed and a continuous 20 MeV proton beam, which delivered a dose of 3 Gy to a HeLa cell monolayer within < 1 ns or 100 ms, respectively. Investigated endpoints were G2 phase cell cycle arrest, apoptosis, and colony formation.

**Results:**

At 10 h after pulsed irradiation, the fraction of G2 cells was significantly lower than after irradiation with the continuous beam, while all other endpoints including colony formation were not significantly different. We determined the relative biological effectiveness (RBE) for pulsed and continuous proton beams relative to x-irradiation as 0.91 ± 0.26 and 0.86 ± 0.33 (mean and SD), respectively.

**Conclusions:**

At the dose rates investigated here, which are expected to correspond to those in radiation therapy using laser-driven particles, the RBE of the pulsed and the (conventional) continuous irradiation mode do not differ significantly.

## Background

Because of the superior dose distribution of protons and heavy ions, radiotherapy using charged particles has attracted increasing interest over the last years [1,2]. At the same time, however, a vivid discussion has started as to whether the potential improvements in outcome justify the costs of particle therapy, where the costs per fraction are estimated to be up to 5 times higher than those for photon therapy [3]. With the advent of ultrafast high energy lasers, the idea of laser-driven acceleration of particles suitable for therapeutic applications has arisen, combined with the hope for a reduction of costs and required space [4-6]. While early concepts may have been a bit over-enthusiastic [7], recent feasibility studies still see a potential for laser-acceleration in radiation therapy [8-10], although the energies achieved at present are far from those required for radiation therapy and many questions remain unresolved, e.g. regarding energy selection, beam preparation and transport, as well as repetition rate.

With respect to potential differences in the radiobiological effects of laser-accelerated particles and those accelerated conventionally by cyclotrons or synchrotrons, the main difference is that particle beams delivered from laser acceleration will be pulsed. While the laser pulses required for the acceleration of high energy particles are in the range of femtoseconds, the particle pulse thus created will spread in time during beam transport. For example, assuming protons with a mean energy of 100 MeV and an energy spread of 1% which are transported over a 20 m distance, the expected duration of the pulse at the target will be about 1 ns [11]. Since the repetition rates of laser accelerators are expected to be rather moderate, one can envision that during one session each voxel of the PTV (planning treatment volume) can be targeted at most a few times if the treatment duration is to be kept reasonably short. Thus, with one pulse a considerable fraction of the required dose at a target voxel has to be given. Assuming a deposition of >1 Gy in 1 ns, this translates to an ultra high dose rate of >10^9 ^Gy s^-1^.

In the past, radiobiological effects of x-ray or electrons delivered at ultra high dose rates were reported: Several authors described an enhanced resistance of cells irradiated with several Gy at dose rates in the range of 10^9 ^Gy s^-1 ^at low oxygen concentrations [12,13] which was ascribed to oxygen depletion. Radical-radical recombination was also proposed as a possible explanation for reduced efficiency of pulsed irradiation [14]. It should be noted that these previous experiments were performed with conventional acceleration. Currently available laser accelerators have so far allowed for the performance of a few proof-of-principle experiments with laser plasma-generated X-rays [15,16] and ultra-soft X rays [17]. Tillmann and coworkers [15] applied a dose of only 3.4 mGy per 2 ps pulse, which at repetition rates of 10 Hz lead to effective dose rates in the range of 2 Gy min^-1^. Hill and coworkers [17] achieved 0.07 Gy per 10 ps pulse, i.e. with 7 × 10^9 ^Gy s^-1 ^a dose rate where high dose rate effects may occur. Finally, Shinohara and coworkers [16] achieved up to 8 Gy per single sub-ps pulse, i.e. relevant dose rates of 10^12^-10^13 ^Gy s^-1^. None of these authors found indications for significant effects of the dose rates associated with laser plasma-generated X-rays as compared to conventional irradiations on cell survival. However, the photon beams used for comparison differed in all three cases in terms of mean energy and energy distribution, so that in the case of small alterations it would be difficult to determine if these are due to differences in dose rate or in energy spectrum. Recently, also proof-of-principle experiments with laser driven protons or electrons were performed, in which it was shown that these beams generate DNA double-strand breaks, as would have been expected [18-20]. A quantitative evaluation and comparison with conventionally accelerated protons was, however, not provided.

In order to simulate the pulsed irradiation conditions expected for laser-accelerated protons in therapy settings, we have established a pulsed proton beam at the ion microbeam SNAKE at the Munich tandem accelerator [11]. By focusing a bunch of 10^5 ^p+ onto a spot of approximately 100 μm × 100 μm we apply macroscopic doses of a few Gy within <1 ns. The irradiations are performed with monoenergetic protons, which enables us to perform irradiations with conventional dose rates for direct comparison with the same beam quality. Using this system, in previous work we reported that the induction of micronuclei in HeLa cell monolayers or in keratinocytes within 3D tissue is not significantly different between pulsed and conventional proton irradiation [21,22]. In an analysis of chromosome aberrations, however, we found evidence that proton irradiation in the pulsed mode may be slightly less effective than in the continuous irradiation mode [23]. Here we extend these studies to the analysis of radiation induced cell cycle arrests and apoptosis. In addition, and most importantly, we also investigate the clonogenic survival of cells irradiated under both conditions. This endpoint is generally considered to be of utmost relevance when judging irradiation conditions.

## Methods

### Cell culture for irradiation

HeLa cells (subtype HeLa-RIKEN) were cultured in RPMI ^TS ^1640 medium (PAA), supplemented with 10% fetal bovine serum, 2 mM L-glutamine, 100 U/ml penicillin, and 50 μg/ml streptomycin in a humidified incubator (5% CO_2_, 37°C). Twelve hours prior to irradiation, cells were seeded in stainless steel cell containers designed for irradiation at SNAKE [24]. In these containers, cells grow on a 6 μm thick Mylar foil (carrier foil), covered with CellTAK™ Tissue Adhesive (BD Biosciences) for better attachment. In order to provide restricted growth areas in these containers, two different cylindrical adapters can be mounted onto the container. The growth areas thus obtained are 3.14 cm^2 ^for 2 × 10^5 ^cells in immunofluorescence experiments, or 0.65 cm^2 ^for 3 × 10^4 ^cells in clonogenic survival experiments. Since the irradiation setup at SNAKE requires a vertical sample position, the adapters were removed from the incubator directly before treatment and the containers were sealed with another Mylar foil glued to a stainless steel lid. During irradiation, which takes less than 10 min, the cells are not covered by medium, but a medium reservoir ensures a humid atmosphere. After irradiation cells were given fresh medium and they were incubated for 0 to 48 hours until fixation for immunofluorescence. For clonogenic survival experiments the cells were harvested one hour after radiation treatment and appropriate numbers of cells were reseeded in 6-well tissue containers. The same cell cultivation procedures and same containers were used in proton irradiation and X-ray experiments.

### Irradiation with 250 kV X-rays

X-ray irradiation was performed with a Philips MCN-ray tube (250 kV, 13 mA, 2.5 + 4.0 mm Al and 1.0 mm Cu filtration) at a dose rate of 0.56 Gy min^-1^. HeLa cells grown on Mylar foil were irradiated with doses from 0 to 5 Gy for a reference survival curve (2 independent experiments) and with 3 Gy for immunofluorescence detection of cell cycle arrest and apoptosis (5 to 7 independent experiments, each with a non-irradiated control).

### Irradiation with pulsed and continuous proton beams

Proton irradiation was performed using the microprobe SNAKE at the Munich tandem accelerator in a pulsed and a continuous irradiation mode. Beam preparation and dosimetry [11], as well as cell irradiation with pulsed and continuous proton beams at SNAKE [21] were performed as described previously. For pulsed irradiations, about 10^5 ^protons were focused onto a beam spot of approximately 100 × 100 μm^2^, which was then scanned with 100 Hz over a region of 1.5 × 2 mm^2 ^for immunofluorescence staining experiments. This scanning results in a nearly homogeneous dose distribution, where between 25% and 60% (on average nearly 50%) of the total dose (3 Gy) to each cell was given by a single < 1 ns pulse, corresponding to an instantaneous dose rate of > 10^9 ^Gy s^-1^, while the remaining dose resulted from contributions of adjacent irradiation fields. In colony formation experiments larger irradiation fields were required in order to entirely cover the circular cell cultivation area of 0.65 cm^2^. Therefore, a region of approximately 10 mm × 10 mm was irradiated by arranging several 1.5 × 2.0 mm^2 ^fields next to each other by moving the cell sample mechanically. In irradiations with the continuous beam, a rectangular beam spot with a size of approximately 2 × 2 mm^2 ^was used, and the time required to deliver the total dose to cells was in the order of 100 ms (dose rate ~30 Gy s^-1^).

Experiments for detection of cell cycle arrest and apoptosis were performed with 20 MeV protons (LET in water 2.648 keV µm^-^^1^. For colony formation, the proton energies were varied between 20 and 25 MeV between experiments for technical reasons. Dose was changed by adjusting the proton fluence accordingly. The variation in LET (between 2.648 keV μm^-1 ^and 2.207 keV um^-1^) was considered to be negligible.

Parallel irradiation experiments with pulsed and continuous beams were always conducted within the same beam time, but for technical reasons they had to be separated by 2-3 days, i.e. the time needed to switch from pulsed to continuous beam preparation.

### Immunofluorescence detection of cell cycle arrest and apoptosis

For immunofluorescence analyses, cells were fixed with 2% paraformaldehyde (PBS-buffered; 15 min) immediately after the irradiation experiment (i.e. on average about 5 min after irradiation, due to the duration of the irradiation and the sample handling) or after incubation for 10, 24, or 48 hours. Permeabilisation (0.15% TritonX 100 in PBS) and blocking (1% BSA, 0.15% Glycine in PBS) were done as described previously [24]. Epifluorescence microscopy was performed with a Zeiss AxioObserver Z1 inverse epifluorescence microscope (Germany), using a Zeiss LCI Plan Neofluar 63/1.3 objective, the software AxioVision 4.6 and an AxioCam Mrm camera (Zeiss). All images were further processed using the software ImageJ 1.37c (http://www.uhnresearch.ca/wcif).

Cells in G2/M phase were identified by immunostaining with mouse anti-CyclinB1 antibody (dilution 1:100, Abcam #ab49215). G2-specific staining was in initial experiments verified by co-staining with rabbit anti-CENP-F antibody (1:500, Novus Biologicals, #NB500-101), which identifies cells in S/G2/M phase. In cell samples irradiated with protons, the irradiated region was detected by co-staining with rabbit anti-γ-H2AX antibody (1:200, Abcam, # ab11174). After application of fluorescence conjugated secondary antibodies sheep-anti-mouse Cy3 (1:500, Dianova, #515-165-062) and goat-anti-rabbit Alexa488 (1:200, Molecular Probes, #A-11034), DNA was stained with DAPI. The fraction of CyclinB1 positive cells (excluding mitotic cells as identified by chromatin structure), was separately determined for the irradiated area and a non-irradiated area (n. i.) of the same sample. The data presented give the mean and standard error of the mean (SEM) of 3 independent proton experiments performed within 3 different beam times and 5 to 7 independent x-ray experiments.

Apoptotic cells were initially identified by staining with rabbit anti-cleaved caspase3 (1:100, Trevigen, #2305-PC-050) and goat-anti-rabbit Alexa488 (1:200, Molecular Probes, #A-11034). DNA was stained with DAPI. Since the same results were obtained when identifying apoptotic cells based on the characteristic chromatin structure after DAPI staining, in later experiments the apoptotic cells were identified simply by chromatin structure. In samples irradiated with protons, the irradiated area was detected by immunostaining with rabbit anti-γ-H2AX antibody (1:200, Abcam, # ab11174). The percentage of apoptotic cells was determined in 2 to 5 proton experiments per point of time, performed within 4 different beam times, and 5 to 7 independent x-ray experiments (mean and SEM).

### FACS analysis of cell cycle distribution

For quantitation of cells in G2/M phase by FACS analysis, cells were harvested 10 and 24 hours after irradiation with x-rays (3 Gy and sham-irradiated controls) and washed with PBS. Cells were resuspended in 0.5 ml DNA-staining solution I (PBS containing 10 μg/ml RNAse, 0.6 mg/ml NaCl, 1 mg/ml Sodium citrate, 0.07% Nonidet^® ^P 40 Substitute, 20 μg/ml propidium iodide (PI)) and incubated at room temperature in the dark for 30 min, before 0.5 ml of ice-cold DNA-staining solution II (PBS containing 15 μg/ml citric acid, 85 μg/ml sucrose, 20 μg/ml PI) was added. Samples were stored at 4°C until PI-detection with BD FACSCanto™. Data were analysed with BD FACSDiva™ software.

### Clonogenic survival

About 24 h prior to irradiation, 3 × 10^4 ^HeLa cells were seeded in irradiation containers on Mylar foil, restricted to an area of 0.65 cm^2 ^by a special cylindrical adapter. To characterize survival after irradiation with pulsed and continuous protons, we have chosen a dose of 3 Gy, analogous to the other investigated endpoints. For creating a reference survival curve, cells were irradiated with 250 kV x-rays, with doses ranging from 0 to 5 Gy. One hour after irradiation, cells were harvested by trypsination and counted with a Bürker chamber. Cells were reseeded in 3 different titres and in triplicates into 6-wells containers. The cells were maintained at 37°C and 5% CO_2 _for 10 days to allow colony formation. After this time period, the cells were fixed and stained with ethanol (80%) containing 0.3% methylene blue for 30 minutes. After washing, the samples were air-dried and microscopically examined. Colonies consisting of more than 50 cells were considered as survivors. The plating efficiency (PE = cells seeded/colonies counted) was calculated for non-irradiated cells and the surviving fraction of irradiated cells (SF = [cells seeded/colonies counted]/PE) was determined. Each experiment was performed at least twice and mean and standard deviation (SD) are presented.

The RBE of protons (pulsed or continuous) is calculated as the ratio between the dose, Dγ, of the reference radiation (250 kV X-rays) and the dose, Dp, of protons which produced equal response, y: RBE = Dγ/Dp. To calculate Dγ the measured dose response curve is parameterised by a linear-quadratic function y = c + αD+ βD2, fitted to determine the parameter c, α and β and inverted.

As the fit parameters, c, α and β, depend on each other, a Monte Carlo simulation-based Bayesian data analysis is performed. In a first step the three-dimensional probability density is calculated as a function of c, α and β for measuring the data set used for the dose response curve. In a second step, assuming a Gaussian probability density for y, the probability density for Dγ is calculated leading to the confidence interval for Dγ. From this and from the error of the proton dose measurement the error of the RBE value is calculated by Gaussian error propagation.

## Results

### Experimental Set-up

In order to apply macroscopic doses of a few Gy within 1 ns, we use a proton microbeam facility with which 10^5 ^p+ can be focused onto a spot of approximately 100 μm × 100 μm and delivered within 0.9 ns [11]. This beam spot is scanned over a larger area to irradiate a sufficient number of cells. In our first experiments, the irradiated field was for technical reasons limited to 1.5 × 2 mm^2^. Since this irradiated field is surrounded by a large area (diameter ~3 cm) of unirradiated cells, only biological endpoints amenable to a microscopic evaluation could thus be investigated. We chose microscopic analysis of cell cycle distribution and of apoptosis induction. The irradiated field is identified by concomitant immunofluorescence detection of DNA damage markers such as γ-H2AX or 53BP1.

In later experiments, including experiments on colony formation ability, the field size was increased to 10 × 10 mm^2 ^by introducing additional mechanical scanning of the cell sample. Analysis of colony formation required that all cells on the dish were irradiated before harvest and reseeding. We therefore devised specific cylindrical adaptors with which the growth field on the Mylar foil could be clearly defined. Cells were seeded within this cylinder (9 mm diameter) and allowed to grow and form a sub-confluent layer there. Immediately before the irradiation, the cylinders were removed. For irradiation, the cell field was aligned to the beam by using an optical microscope.

### Cell cycle arrest

Cyclin B1 expression begins in S phase and peaks in G2 phase. Prior to M phase, cyclin B1 is predominantly located in the cytoplasm; after centrosome separation it accumulates in the nucleus, before it is degraded at the transition of meta- to anaphase. Radiation-induced G2 arrest and relative cyclin B1 expression were shown to correlate well in Hela cells [25]. To test the correlation between DNA content and microscopically detectable cyclin B1 localization in cytoplasm or nucleus, parallel samples were subject to PI staining and flow cytometry, or to immunofluorescence with cyclin B1 staining (Additional file 1). A clear accumulation of G2/M phase cells is detected 10 h after irradiation (3 Gy x-ray) in irradiated cells, while after 24 h the percentage of G2/M cells in irradiated samples returned to a level similar to unirradiated controls. When mitotic cyclin B negative cells, which were identified microscopically by the typical chromatin appearance, were added to the numbers of cyclin B1 positive cells, both methods gave very similar results, demonstrating that microscopic determination of cyclin B1 staining can be used to determine G2 phase cells.

**Figure 1:**
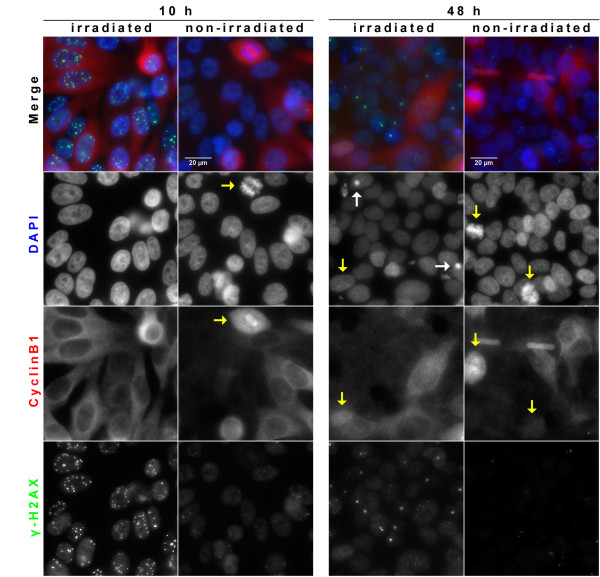
**Detection of G2 phase cells by cyclinB1 staining**. Immunofluorescence detection of cyclin B (red) 10 h (left) and 48 h (right) after continuous proton irradiation (3 Gy) and sham irradiation. Irradiated cells are identified by γ-H2AX staining (green). Apoptotic cells (white arrows) and mitotic cells (yellow arrows) are identified by their appearance after DAPI staining (blue).

**Figure 2:**
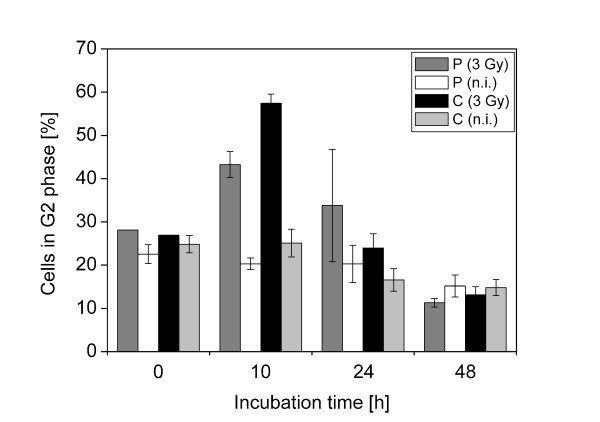
**Accumulation of cells in G2 phase after irradiation**. Cells were irradiated with protons (3 Gy) in pulsed mode (P) or continuous mode (C). Data are from 3 independent experiments, except for irradiated (3 Gy) sample at 0 h (1 experiment). Indicated are means and standard errors of the mean (SEM).

To compare the induction of G2 arrest in cells irradiated by 3 Gy pulsed and continuous proton irradiation, the cells were irradiated under the respective conditions, and fixed at the earliest time point amenable (on average about 5 min) or, after removal of the lid and medium exchange, incubated for 10 h, 24 h, or 48 h at 37°, 5% CO_2 _before fixation. Samples were simultaneously stained for cyclin B1 to determine the cell cycle position and γ-H2AX to identify the irradiated field. Typical examples are shown in Figure 1. The proportion of G2 phase cells (defined as cyclin B1 positive, without mitotic cells) among cells exhibiting damage markers was determined by manual counting of at least 130 cells per experiment. After proton irradiation the maximum G2 phase arrest is evident after 10 h, while after 24 h the proportion of G2 phase cells is similar to the situation before irradiation (Figure 2). Interestingly, at 10 h after irradiation the proportion of G2 phase cells after continuous proton irradiation was found to be significantly higher than after pulsed irradiation (t-test, two-tailed p = 0.0182). To test whether the difference can be explained by small differences in absolute dose between the samples irradiated at pulsed and continuous mode, we asked whether a substantial increase in dose would affect the outcome and compared the induction of G2 phase arrest after x-irradiation of HeLa cells with 3 and 5 Gy (Additional file 2). Since the fraction of G2 phase cells after 10 h is practically the same at both doses, we conclude that the differences between pulsed and continuous mode cannot be explained by small variations in dose. We note that the duration of the G2 phase arrest appears to be influenced by dose.

### Radiation induced apoptosis

**Figure 3:**
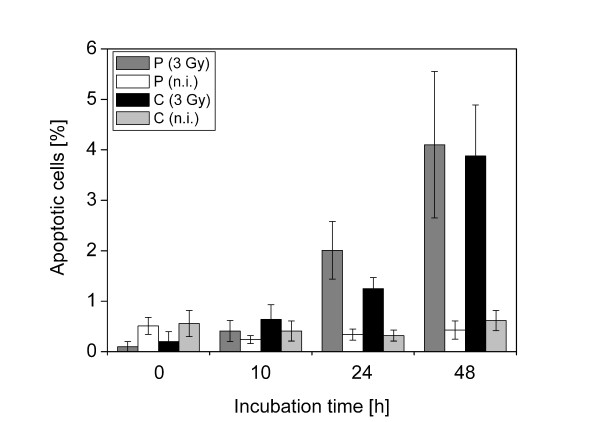
**Induction of apoptosis after irradiation**. Cells were irradiated with protons (3 Gy) in pulsed mode (P) or continuous mode (C). Data are from 5 independent experiments except for data at 10 h (4 experiments) and irradiated samples at 0 h (2 experiments). Indicated are means and standard errors of the mean (SEM).

Since the number of samples that can be irradiated at SNAKE is very limited, we aimed at determining the induction of apoptosis in the samples used for determination of G2 arrest. In preliminary experiments we verified by co-staining with an antibody recognizing the apoptosis indicator, cleaved caspase 3, that apoptotic cells can be identified after DAPI staining by the appearance of bright circular bodies (Additional file 3). After irradiation with x-rays, the proportion of apoptotic cells increases with time, but the inter-experimental variation is very high (Additional file 4). One likely explanation is that in the course of the immunofluorescence detection, which involves several washing steps, apoptotic cells can easily be lost. Comparing induction of apoptosis after proton irradiation in the continuous mode and the pulsed mode, no significant difference was seen (Figure 3).

### Colony formation

**Figure 4:**
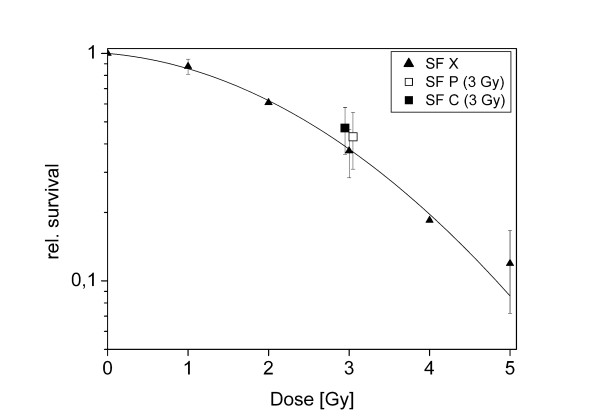
**Colony formation after irradiation with pulsed and continuous proton beams**. Indicated is relative survival (surviving fraction, SF) of HeLa cells after irradiation with pulsed (SF P, open square) or continuous (SF C black square) proton beams at 3 Gy. Mean and SD of 3 independent experiments in 3 different beam times. Reference: x-ray irradiation (SF X, black triangles), mean and SD of 2 independent experiments.

Colony formation is one of the most important endpoints in studying radiobiological effects. After irradiation with 3 Gy using pulsed and continuous proton beams, the fraction of colony forming cells was 0.43 ± 0.07 (mean and SEM) and 0.47 ± 0.06, respectively. This difference is not statistically significant. For comparison the survival curve after x-irradiation is also shown (Figure 4). We determined the relative biological effectiveness (RBE) for pulsed and continuous proton beams relative to x-irradiation as 0.91 ± 0.26 and 0.86 ± 0.33 (mean and SD), respectively. These values are not significantly different.

## Discussion

The present work is part of a series of systematic comparisons of biological endpoints after irradiation with pulsed and continuous proton beams, which in their dose rate differ by a factor of 10^8^. Using Monte Carlo-based modelling, Kreipl et al. conducted a systematic analysis of the impact of spatial and temporal proximity of ion tracks on the yield of hydroxyl radicals [26]. While indeed the yield of hydroxyl radicals is expected to be reduced when two ion tracks overlap closely in time and space, the authors conclude that at typical doses associated with radiation therapy (2 - 5 Gy), the spatial separation of two tracks is larger than would be necessary for the reduction in hydroxyl radical yield to occur. This holds for proton tracks, and even more so for heavier ions. Indeed, in our previous analyses we did not detect statistically significant differences between both irradiation modes when investigating a variety of specific endpoints related to chromosomal damage, i.e. micronuclei and chromosomal aberrations [21-23]. We consistently observed, however, a slightly reduced apparent efficiency of the pulsed beam in inducing the damage compared to the continuous beam. When combining data for different types of chromosomal aberrations (dicentrics, centric rings and excess acentrics) and thus increasing the sample size, we recently observed a statistically significantly reduced effectiveness of pulsed irradiation [23].

Here, we report for the first time a reduced effectiveness of the pulsed irradiation mode on a single endpoint, namely G2 phase arrest at 10 h after irradiation (Figure 2), thus substantiating the previous observations. This difference is somewhat surprising, given that the fraction of cell arresting in G2 appears to be rather insensitive to dose, at least in the dose range of 3 to 5 Gy x-irradiation (Additional file 2). While exhibiting a high degree of variation, we find indications that the duration of the G2 arrest may be longer after irradiation in the pulsed mode than in the continuous mode (Figure 2), possibly hinting at a shift in the time to maximum G2 arrest and in duration. Further experiments are necessary to clarify this point. We cannot exclude at present that the observed difference reflects differences in damage complexity, as it is well known that the duration of the G2 arrest is affected by LET [27]. There is no evidence that systematic errors in dosimetry had influence on this result, because the same setup was used for preparation of the pulsed and the continuous beam for proton irradiation.

In any case, the difference in cell cycle response did not translate into a difference in colony forming ability (Figure 4). While the pulsed beam was slightly more efficient in cell killing, the difference was not significant. With 0.91 ± 0.26 and 0.86 ± 0.33 for pulsed and continuous mode, respectively, and considering that x-rays were used as reference irradiation rather than gamma rays, the RBE determined for both irradiation modes is compatible with the generally assumed RBE of 1.1, which is also applied in proton therapy [28]. We cannot exclude at the moment that at even higher dose rates in pulsed mode, differences would become apparent.

While production of ultra high dose rate pulses emerges as an interesting new application of microbeams that can even be used for irradiation of mice [29,30], the dose rates achievable with our set-up are limited. Experiments to investigate the effects of laser-driven protons, which would deliver their dose in the ps range if the distance between proton source and cell targets is not too large, are currently under way in our laboratory. It is important to note, however, that in a clinical application of laser-driven protons, the proton source is also expected to be not very close to the patient, since high levels of unspecific dose distributions from primary and secondary neutron and high energy x-ray fields and the need for selection of protons with appropriate energy from the broad energy distribution typically obtained by laser acceleration will require a beam line which for radiation protection reasons may not be much shorter than the beam lines known from conventional acceleration. We currently estimate the pulse duration at the tumor voxel in the range of 1 ns [11].

## Conclusion

At the dose rates investigated here, which are expected to correspond to those in radiation therapy using laser-driven particles, the RBE of the pulsed and the (conventional) continuous irradiation mode do not differ significantly.

## List of abbreviations

DAPI: 4',6-Diamidin-2-phenylindol; FACS: Fluorescence-activated cell sorter; LET: linear energy transfer; RBE: relative biological effectiveness; SEM: standard error of the mean; SD: standard deviation

## Competing interests

The authors declare that they have no competing interests.

## Authors' contributions

SA performed and evaluated most of the biological experiments. VH, CG and GD performed the microbeam irradiations. GAD helped with beam time organization and experiments. TES helped with beam time organization and statistical evaluation of colony formation experiments. CB and GD contributed to study design and manuscript preparation. AAF was the Principal Investigator of the study and prepared the manuscript. All authors read and approved of the manuscript.

## Supplementary Material

Additional file 1 **Agreement of quantitative determination of G2/M phase cells by FACS analysis and microscopy**. This figure shows the results of a quantitative evaluation of the number of G2/M phase cells determined by FACS analysis and microscopy in parallel samples. Click here for file

Additional file 2 **Dose-dependence of accumulation of cells in G2 phase after x-irradiation**. This figure shows the frequency of cells in G2 phase after irradiation with 0, 3 and 5 Gy and incubation for 0, 10, 24 and 48 h. Click here for file

Additional file 3 **Microscopic identification of apoptotic cells**. This figure shows examples of apoptotic cells identified by staining for cleaved caspase 3 and by appearance after DAPI staining. Click here for file

Additional file 4 **Dose-dependence of induction of apoptotic cells after x-irradiation**. This figure shows the frequency of apoptotic cells after irradiation with 0, 3 and 5 Gy and incubation for 0, 10, 24 and 48 h. Click here for file
