# Supplementary material for: Survival of tumor cells after proton irradiation with ultra-high dose rates
Source: Radiat Oncol. 2011 Oct 18;6:139. doi: 10.1186/1748-717X-6-139 (PMC3215966; doi:10.1186/1748-717X-6-139)
Supplement: Additional file 1 — Agreement of quantitative determination of G2/M phase cells by FACS analysis and microscopy. This figure shows the results of a quantitative evaluation of the number of G2/M phase cells determined by FACS analysis and microscopy in parallel samples. [file 1748-717X-6-139-S1.PDF]

Auer et al.: Additional file 1

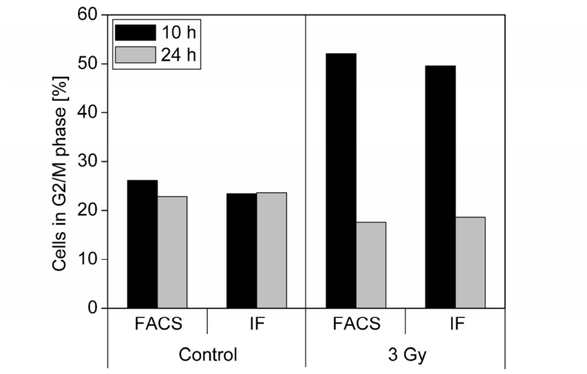

Additional file 1. Agreement of quantitative determination of G2/M phase cells by FACS analysis and microscopy. Microscopic evaluation was performed after immunofluorescence staining for cyclin B. Mitotic cyclin B-negative cells (0 Gy 10 h: 5.6%; 0 Gy 24 h: 3.2%; 3 Gy 10 h: 0.4%; 3 Gy 24 h: 3.2%) were added to the number of cyclin B-positive cells in order to obtain the fraction of G2/M cells via microscopy. Data are from one experiment with parallel samples irradiated with x-rays (3 Gy) or sham-irradiated, and harvested at 10 h or 24 h after irradiation.
