# Supplementary material for: Survival of tumor cells after proton irradiation with ultra-high dose rates
Source: Radiat Oncol. 2011 Oct 18;6:139. doi: 10.1186/1748-717X-6-139 (PMC3215966; doi:10.1186/1748-717X-6-139)
Supplement: Additional file 2 — Dose-dependence of accumulation of cells in G2 phase after x-irradiation. This figure shows the frequency of cells in G2 phase after irradiation with 0, 3 and 5 Gy and incubation for 0, 10, 24 and 48 h. [file 1748-717X-6-139-S2.PDF]

Auer et al.: Additional file 2

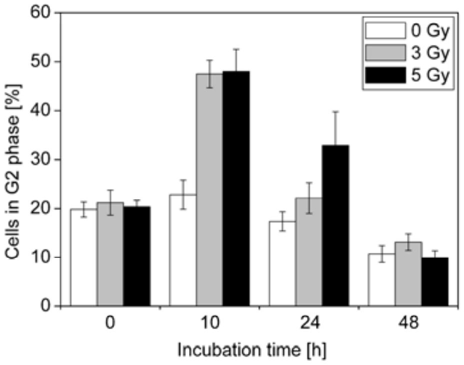

Additional file 2. Dose-dependence of accumulation of cells in G2 phase after x-irradiation. Cell were irradiated with 3 Gy and 5 Gy. Data are from 5 to 7 independent experiments. Indicated are means and standard errors of the mean (SEM).
