# Supplementary material for: Survival of tumor cells after proton irradiation with ultra-high dose rates
Source: Radiat Oncol. 2011 Oct 18;6:139. doi: 10.1186/1748-717X-6-139 (PMC3215966; doi:10.1186/1748-717X-6-139)
Supplement: Additional file 3 — Microscopic identification of apoptotic cells. This figure shows examples of apoptotic cells identified by staining for cleaved caspase 3 and by appearance after DAPI staining. [file 1748-717X-6-139-S3.PDF]

Auer et al.: Additional file 3

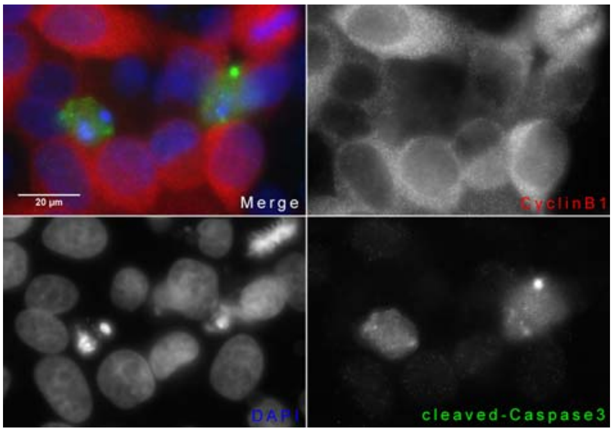

Additional file 3. Microscopic identification of apoptotic cells. Cells were identified after immunofluorescence staining for cleaved caspase 3 (green) and by appearance after DAPI staining (blue). Note the differential DAPI morphology of apoptotic cells and mitotic cells (upper right corner; the mitotic cell is identified by nuclear staining for cyclin B1 (red)).
