# Supplementary material for: Survival of tumor cells after proton irradiation with ultra-high dose rates
Source: Radiat Oncol. 2011 Oct 18;6:139. doi: 10.1186/1748-717X-6-139 (PMC3215966; doi:10.1186/1748-717X-6-139)
Supplement: Additional file 4 — Dose-dependence of induction of apoptotic cells after x-irradiation. This figure shows the frequency of apoptotic cells after irradiation with 0, 3 and 5 Gy and incubation for 0, 10, 24 and 48 h. [file 1748-717X-6-139-S4.PDF]

Auer et al.: Additional file 4

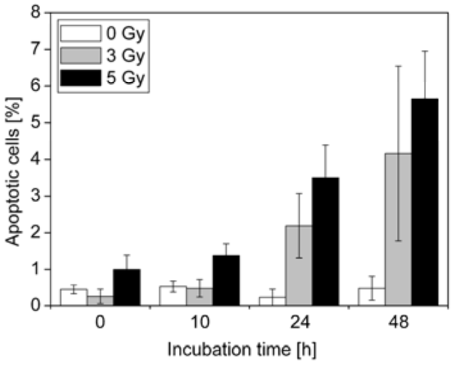

Additional file 4: Dose-dependence of induction of apoptotic cells after x-irradiation. Cells were irradiated with 3 Gy and 5 Gy. Indicated are mean and SEM from 5 to 7 independent experiments.
